# Virtual reality surgical simulator training of residents and early-career surgeons for pedicle screw placement: a systematic review and meta-analysis

**DOI:** 10.1007/s00590-026-05008-1

**Published:** 2026-10-27

**Authors:** Eric Pasqualotto, Tales Pasqualotto, Victor Hugo Pinheiro Lopes, Laura Lunelli, Lucas Loiola Santos

**Affiliations:** 1 Programa de Pós-Graduação em Cirurgia Translacional, Universidade Federal de São Paulo, São Paulo, Brazil; 2 Departamento de Medicina, University Alto Vale do Rio do Peixe, Caçador, Brazil; 3 Departamento de Medicina, Universidade Federal do Ceará, Fortaleza, Brazil; 4 Departamento de Medicina, Universidade do Sul de Santa Catarina, Palhoça, Brazil; 5 Departamento de Cirurgia, Divisão de Neurocirurgia, Servidores do Estado University Hospital, Federal University of Rio de Janeiro State (UNIRIO), Rio de Janeiro, Brazil., Rio de Janeiro, Brazil

**Keywords:** Virtual reality, Training, Pedicle screw

## Abstract

**Purpose:**

To evaluate the effectiveness of Virtual reality (VR) simulation training compared with traditional teaching methods for residents and early-career surgeons training in pedicle screw insertion.

**Methods:**

PubMed, Embase, and the Cochrane Library were systematically searched from inception to December 2025. Randomized controlled trials (RCTs) comparing VR simulator training with traditional training approaches for pedicle screw placement in residents and early-career surgeons were included. A random-effects model was applied to pool outcomes, with treatment effects expressed as mean differences or risk ratios with 95% confidence intervals.

**Results:**

A total of six RCTs were included, with 54 trainees and 457 screws inserted in each study group. Pooled analyses demonstrated that residents and early-career surgeons trained with VR simulation achieved a significantly higher rate of optimal (Grade I) pedicle screw placement than those receiving conventional training (RR 1.36; 95% CI 1.15 to 1.61). Conversely, VR training was associated with significantly lower rates of Grade II (RR 0.61; 95% CI 0.45 to 0.85), Grade III (RR 0.07; 95% CI 0.02 to 0.30), and Grade IV screw placements (RR 0.16; 95% CI 0.03 to 0.90), indicating consistent reductions across all severities of cortical breach. Overall procedural success was significantly higher in the VR group (RR 1.17; 95% CI 1.04 to 1.32). Furthermore, VR-trained residents and early-career surgeons demonstrated shorter screw penetration distances and fewer displaced screws per resident.

**Conclusions:**

These findings indicate that VR simulation training significantly enhances accuracy and safety-related outcomes in resident and early-career surgeons’ pedicle screw placement training.

**Supplementary Information:**

The online version contains supplementary material available at https://doi.org/10.1007/s00590-026-05008-1.

## Introduction

The emergence of virtual reality (VR) technology has marked a paradigm shift in surgical education over the past two decades. Initially limited by low-fidelity graphics and unrealistic interactions, VR simulators have evolved into high-fidelity platforms capable of reproducing complex anatomical structures, procedural workflows, and, in some systems, haptic feedback. This evolution has enabled immersive, interactive, and repeatable training experiences that enhance skill acquisition while maintaining patient safety [1, 2]. VR-based simulation allows trainees to practice surgical techniques in a controlled, risk-free environment, facilitating experiential learning, immediate feedback, and error correction without clinical consequences. As demonstrated across multiple surgical specialties, VR training has been associated with improved anatomical understanding, accelerated learning curves, and increased trainee confidence, supporting its growing integration into residency curricula [1–5].

Pedicle screw placement is one of the most commonly performed and technically demanding procedures in spine surgery, with broad applications in the management of spinal trauma, degenerative diseases, deformities, tumors, and instability [6, 7]. Accurate screw placement is essential to achieve optimal biomechanical fixation, deformity correction, and long-term construct stability. However, due to the proximity of neural and vascular structures to the pedicle, inaccurate placement may result in serious complications, including neurological injury, vascular damage, nonunion, and implant failure [6–9]. Mastery of this technique requires a steep learning curve, traditionally achieved through cadaveric training and supervised operative exposure. In this context, VR simulation offers a compelling adjunct for resident training by enabling repeated practice, spatial orientation enhancement, and procedural rehearsal, potentially improving accuracy and safety before real-world application [1, 7, 9].

Despite the increasing adoption of VR simulators in spine surgery training, robust and consolidated evidence supporting their effectiveness in improving pedicle screw placement performance remains limited. Existing studies are often small, heterogeneous in design, and vary in simulator type, outcome definitions, and assessment methods, making it difficult to draw definitive conclusions [1, 2, 9–11]. Furthermore, uncertainty persists regarding the magnitude of benefit provided by VR-based training compared with conventional educational approaches. Therefore, we conducted a systematic review and meta-analysis to critically evaluate the effectiveness of VR surgical simulation in training residents and early-career surgeons for pedicle screw placement, with a particular focus on accuracy-related outcomes and educational performance metrics.

## Methods

This systematic review followed the Preferred Reporting Items for Systematic Reviews and Meta-Analysis (PRISMA) guidelines [12]. The study protocol was registered in the International Prospective Register of Systematic Reviews (PROSPERO) with registration number CRD420251271462.

### Search strategy and data extraction

PubMed, Embase, and Cochrane Library were systematically searched from inception to December 2025, with the following search strategy: (“virtual reality” OR VR OR “immersive virtual reality” OR simulator* OR “surgical simulator” OR “surgery simulation” OR “simulation training”) AND (“pedicle screw*” OR “pedicle screw placement” OR “pedicle screw insertion” OR “pedicle fixation”) AND (RCT OR random OR randomly OR randomized OR randomised OR randomization OR “Randomized Controlled Trial” OR “Randomized Clinical Trial”). Aiming the inclusion of additional studies, references of the included articles and systematic reviews of the literature were evaluated [13]. Two authors (E.P. and V.H.P.L.) independently extracted baseline characteristics and data outcomes following predefined search criteria: (1) VR Simulator, (2) pedicle screw placement location, (3) accuracy scoring method, (4) number of screws, (5) number of trainees, (6) experience/level of trainees, and (7) VR training duration. Three authors resolved disagreements by consensus (E.P., V.H.P.L., and L.L.S.).

### Eligibility criteria

Studies were included if they met the following criteria: (1) RCTs; (2) comparing VR surgical simulator training with traditional training approaches without VR; (3) enrolling residents and early-career surgeons performing pedicle screw placement; and (4) reporting at least one outcome of interest. We excluded: (1) non-RCTs; (2) overlapping populations; (3) conference abstracts; (4) inclusion of experienced surgeons rather than residents; (5) inclusion of undergraduate medical students; or (6) absence of relevant outcomes related to pedicle screw placement accuracy or performance metrics.

### Endpoints and subgroup analysis

The outcomes of interest were: pedicle screw placement accuracy (Grades I–IV), overall screw placement success, screw penetration distance (mm), and the number of displaced screws per resident.

To harmonize the heterogeneous definitions of pedicle screw accuracy reported across studies, we categorized screw placement into four standardized classes based on the degree of pedicle wall violation. Class I represented ideal placement without cortical violation; Class II represented minor cortical breaches of ≤2 mm or limited pedicle wall violation; Class III represented moderate breaches of approximately 2–4 mm or greater pedicle wall involvement; and Class IV represented severe breaches of ≥4 mm, >50% screw diameter violation, or anterior vertebral wall violation. These categories were mapped to the corresponding grading systems reported in each study, including the Gertzbein–Robbins classification. These definitions are detailed in Supplementary Material 1, Table S1.

### Risk of bias and quality assessment

The Cochrane Collaboration tool for assessing risk of bias in randomized trials (RoB-2) was used to assess the quality of individual RCTs [14]. Each trial received a high, low, or unclear risk of bias score in five domains: randomization process; deviations from the intended interventions; missing outcomes; measurement of the outcome; and selection of reported results. Two independent authors conducted the risk of bias assessment (E.P. and T.P.) and disagreements were resolved unanimously with the senior author (L.L.S.).

Quality of evidence was assessed according to the Grading of Recommendation, Assessment, Development and Evaluation (GRADE) guidelines [15, 16]. Very low, low, moderate, or high-quality evidence grades were designed for the outcomes based on the risk of bias, inconsistency of results, imprecision, publication bias, and magnitude of treatment effects.

### Statistical analysis

The treatment effects for continuous outcomes were compared using weighted mean differences (MDs) and binary endpoints were evaluated using risk ratios (RRs), with 95% confidence intervals (CIs). Heterogeneity was assessed using the Cochrane Q test and I^2^ statistic; *P* value < 0.10 was considered indicative of statistically significant heterogeneity, while I^2^ values were interpreted according to established guidelines [17]. DerSimonian and Laird random-effects models were used for all endpoints [18]. Leave-one-out procedures were used to identify influential studies and their effect on the pooled estimates for the success outcome. The Cochrane Handbook for Systematic Reviews of Interventions was used for data handling and conversion [19]. Statistical analyses were performed using R statistical software, version 4.2.3 (R Foundation for Statistical Computing).

## Results

### Study selection and characteristics

**Fig. 1:**
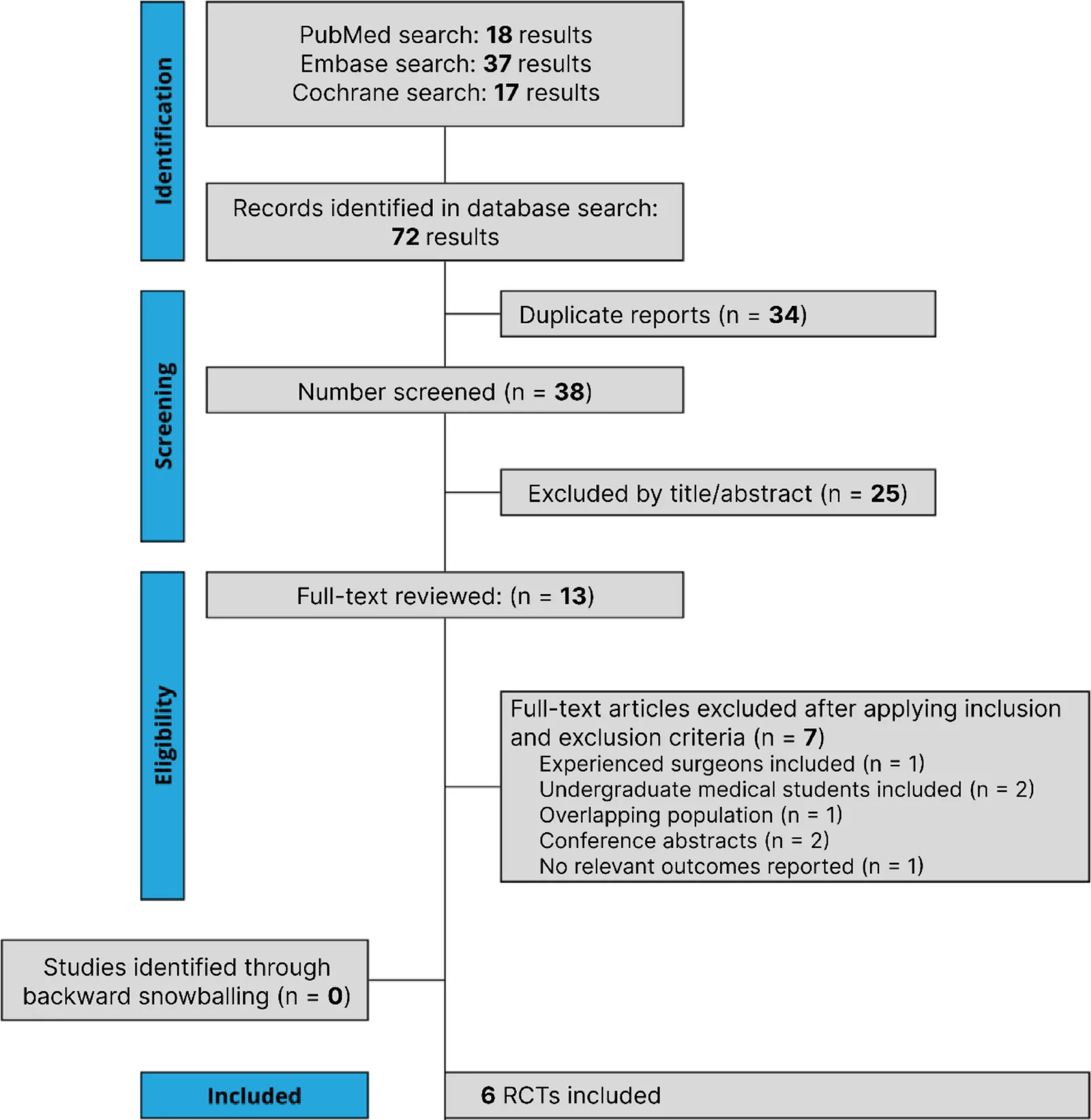
Preferred reporting items for systematic review and meta-analysis (PRISMA) flow diagram of study screening and selection

The initial search yielded 72 results, as detailed in Fig. 1. After removing duplicate records and assessing the studies based on title and abstract, 13 full-text remained and were thoroughly reviewed according to inclusion and exclusion criteria. Of these, 6 RCTs were included comprising 54 trainees and 457 screws inserted in each study group [1, 2, 7, 9–11]. Study and participant characteristics are summarized in Table 1.

**Table 1:** Design and characterístics of studies included in the meta-analysis

Study	VR Simulator	PSP location	Accuracy scoring method	No. of screws VR/Non-VR	No. of trainees VR/Non-VR	Experience/level, n (%)		VR training duration, min‡ (No. of sessions)
						VR	Non-VR	
Alam [1]	ImmersiveSimTM	Lumbar spine sawbone model (L1-L5)	Gertzbein-Robbins assessed by NCCT-scan	19/19*	19/19	8 PGY 1-3 (42.1) 6 PGY 4-6 (31.6) 5 PGY ≥ 7 (26.3)	NR	NR (1)
Hou [7]	VSTS	Fresh adult spine specimens (T6-T12)	CT-based pedicle screw breach grading (4-grade scale)	70/70	5/5	3 PGY 1 (60) 2 PGY 2 (40)	2 PGY 1 (40) 3 PGY 2 (60)	30 (1)†
Hou [11]	VSTS	Fresh adult spine specimens (C3-C6)	Fluoroscopy and CT–based pedicle screw grading (3-grade scale)	40/40	5/5	Residents without experience in PSP		30 (1)
Shi [2]	VSTS	Cadaveric spine specimens (L1-L5)	CT-based pedicle screw breach grading (4-grade scale)	40/40	5/5	Residents without experience in PSP		30 (1)
Xin [10]	IVRSS-PSP	Cadaveric specimens (T11-L4)	CT-based pedicle screw breach grading (4-grade scale)	48/48	8/8	Surgical graduate students		30 (3)
Xin, [9]	IVRSS-PSP	Human patients (thoracic and lumbar spine)	Intraoperative fluoroscopy + Gertzbein–Robbins (CT)	240/240*	12/12	Spine surgeons with less than 1 year of attending experience		40 (5)

CT: computed tomography; IVRSS: immersive virtual reality surgical simulator; NCCT: noncontrast computed tomography; VR: virtual reality; VSTS: virtual surgical training system; NR: not reported; PSP: pedicle screw placement; PGY: postgraduate year; *only post-training screws considered; †15-min VSTS familiarization session not included; ‡time per session

### Pooled analysis of all studies

**Fig. 2:**
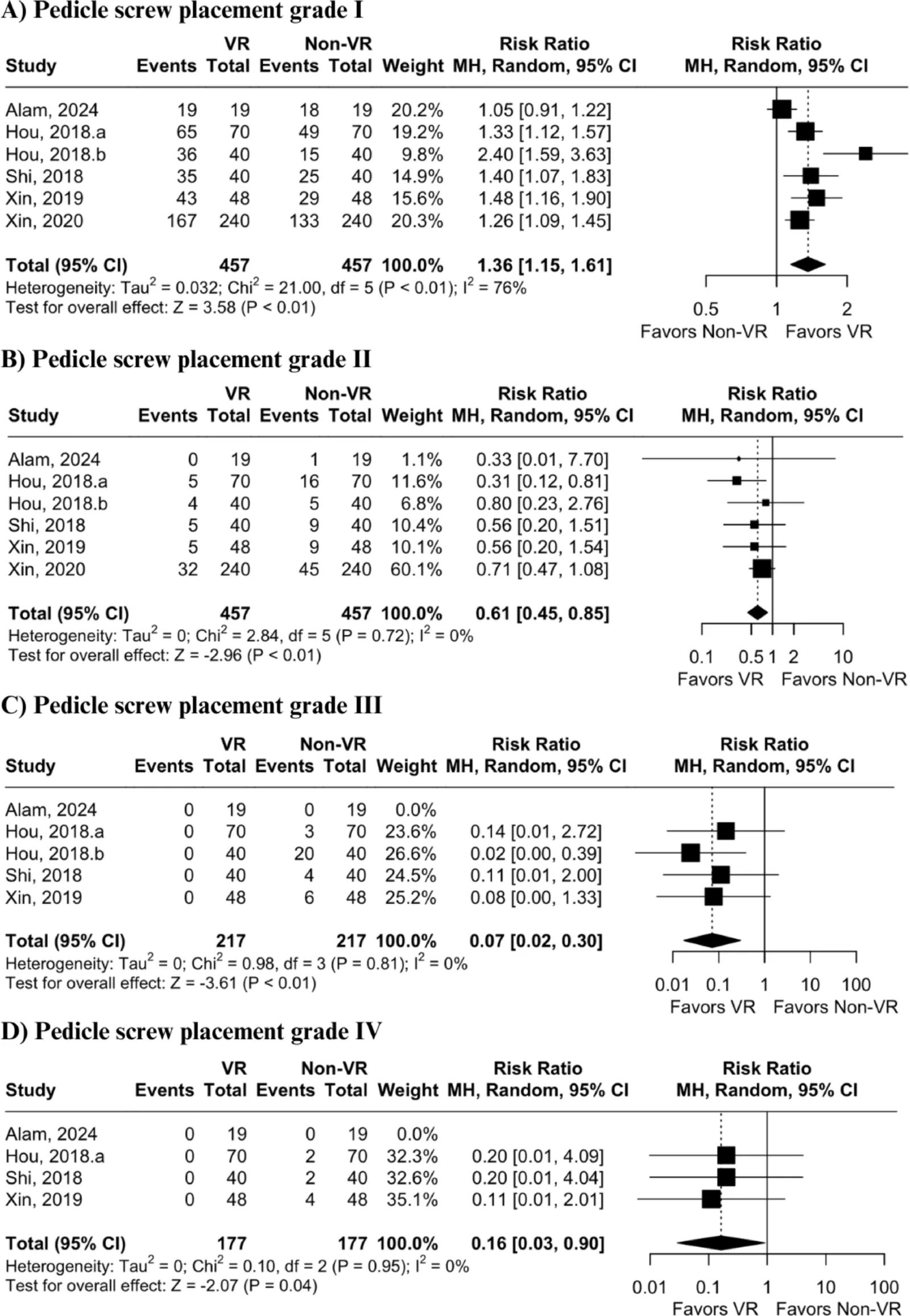
Forest plots of pooled comparisons between virtual reality surgical simulator training versus traditional training approaches for residents and early-career surgeons performing pedicle screw placement. **A** Pedicle screw placement grade I. **B** Pedicle screw placement grade II. **C** Pedicle screw placement grade III. **D** Pedicle screw placement grade IV

**Fig. 3:**
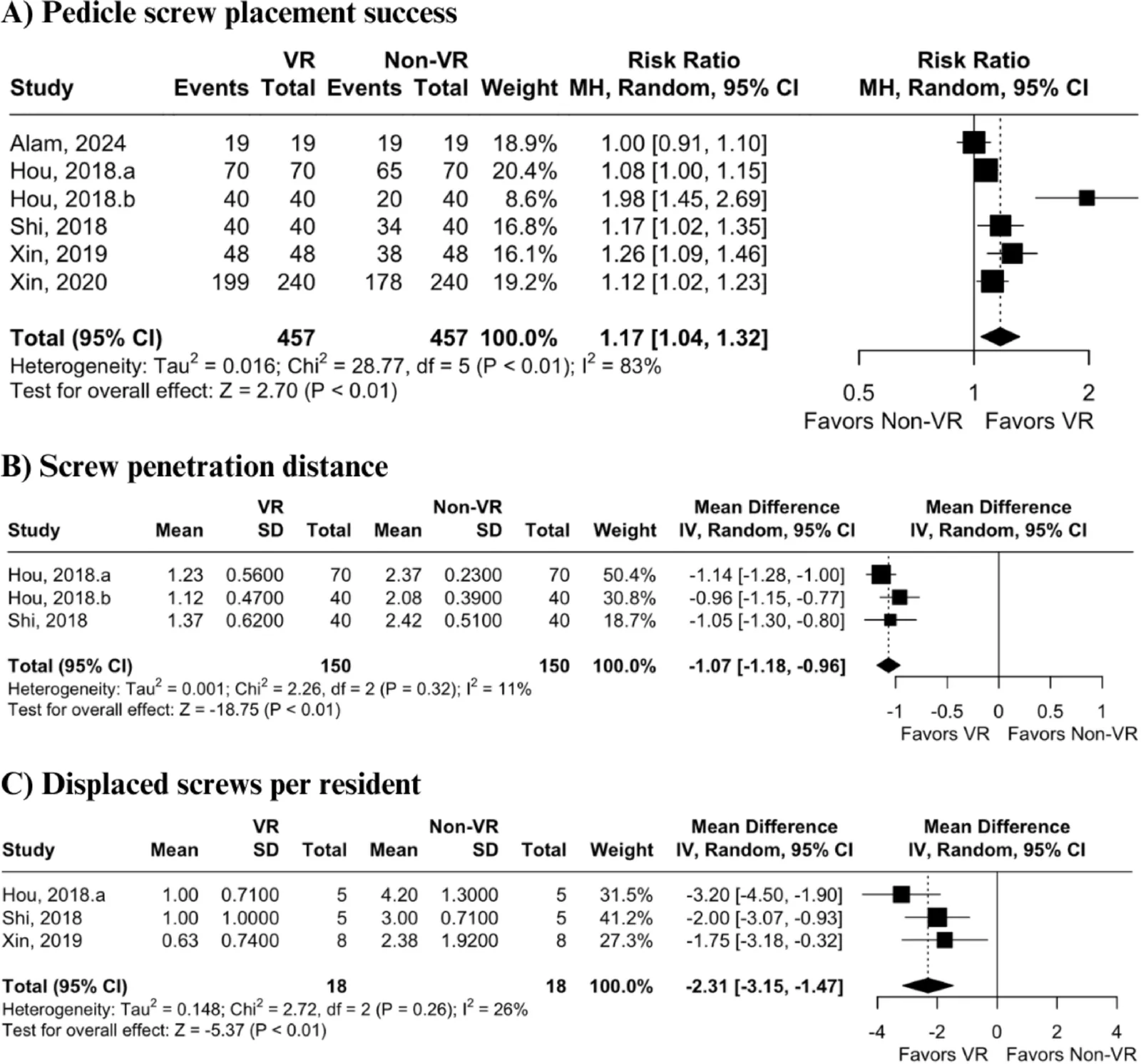
Forest plots of pooled comparisons between virtual reality surgical simulator training versus traditional training approaches for residents and early-career surgeons performing pedicle screw placement. **A** Pedicle screw placement success. **B** Screw penetration distance. **C** Displaced screws per resident

In the pooled analysis, residents and early-career surgeons trained with VR simulation achieved a higher rate of Grade I screw placement compared with traditional approaches (RR 1.36; 95% CI 1.15 to 1.61; *p *< 0.01; I^2 ^= 76%; Fig. 2A). Conversely, VR training was associated with significantly lower rates of Grade II (RR 0.61; 95% CI 0.45 to 0.85; *p *< 0.01; I^2 ^= 0%; Fig. 2B), Grade III (RR 0.07; 95% CI 0.02 to 0.30; *p *< 0.01; I^2 ^= 0%; Fig. 2C), and Grade IV placements (RR 0.16; 95% CI 0.03 to 0.90; *p* = 0.04; I^2 ^= 0%; Fig. 2D), indicating substantial reductions in minor, moderate, and severe cortical breaches. VR-trained residents and early-career surgeons also demonstrated a higher overall success rate (RR 1.17; 95% CI 1.04 to 1.32; *p *< 0.01; I^2 ^= 83%; Fig. 3A).

In the VR training group, there were a significant reduction in screw penetration distances (MD − 1.07 mm; 95% CI − 1.18 to − 0.96; *p *< 0.01; I^2 ^= 11%; Fig. 3B) and fewer displaced screws per resident (MD − 2.31 screws; 95% CI − 3.15 to − 1.47; *p *< 0.01; I^2 ^= 26%; Fig. 3C).

### Sensitivity analysis

We performed a leave-one-out sensitivity analysis for the success outcome. The outcome showed stability, without major changes in significance with the removal of each individual study. The leave-one-out sensitivity analysis plot is detailed in Supplementary Material 1, Fig. S1.

### Risk of bias

Figure S2 presents the detailed risk-of-bias assessment for each RCT included in the meta-analysis. Overall, all studies were rated as having some concerns for risk of bias [1, 2, 7, 9–11].

According to the GRADE assessment, two outcomes were rated as high-certainty evidence: Grade III screw placement and Grade IV screw placement. Three outcomes were rated as low-certainty evidence: Grade II screw placement, screw penetration distance, and displaced screws per resident, while Grade I screw placement and overall pedicle screw placement success were rated as very low-certainty evidence. The main factors responsible for downgrading the certainty of evidence included: the presence of some concerns for risk of bias in all RCTs and high heterogeneity in some outcomes. Quality assessment is detailed in Supplementary Material 2.

## Discussion

In this systematic review and meta-analysis of six RCTs encompassing 54 trainees and 457 screws inserted in each study group, VR–based simulation training was associated with significantly superior pedicle screw placement performance among residents compared with traditional training methods. Residents and early-career surgeons exposed to VR simulation achieved a higher proportion of Grade I screw placements, reflecting optimal intrapedicular positioning, alongside a consistent reduction in malpositioned screws across all breach severity grades, indicating substantial reductions in minor, moderate, and severe cortical breaches. These improvements translated into a higher overall success rate, as well as clinically meaningful reductions in screw penetration distance and the number of displaced screws per resident.

Established principles of surgical education and motor skill acquisition support the observed benefits of VR-based training. VR simulators provide an immersive, three-dimensional environment that facilitates spatial understanding of pedicle anatomy, trajectory planning, and depth perception, key determinants of accurate screw placement [20–22]. Furthermore, the ability to engage in deliberate practice with immediate feedback allows trainees to identify errors and refine technique without the time pressure and cognitive load inherent to the operating room [20–23]. Repeated exposure to complex anatomical scenarios in a risk-free setting may enhance procedural automation and reduce performance variability, leading to improved accuracy during real or simulated screw insertion tasks [20–22].

Our findings are consistent with and extend the results of previous individual studies evaluating VR simulation in spine surgery training. Prior randomized trials have suggested that VR-based systems, including virtual surgical training platforms with or without haptic feedback, may improve pedicle screw accuracy and reduce breach rates compared with conventional teaching approaches [1, 2, 7, 11]. However, these studies were limited by small sample sizes and heterogeneity in outcome assessment. By quantitatively synthesizing available evidence, the present meta-analysis provides a more robust and comprehensive evaluation of the effectiveness of VR training, demonstrating consistent reductions across all grades of screw misplacement severity.

From a clinical and educational perspective, these findings have important implications for spine surgery training programs. Pedicle screw misplacement is associated with potentially severe neurological and vascular complications, and improving technical accuracy at the trainee level may contribute to enhanced patient safety [7, 9]. VR simulation offers a standardized, reproducible, and scalable training modality that can complement traditional educational methods, particularly in settings with limited access to cadaveric models or operative exposure [7]. Incorporating VR-based training into residency curricula may facilitate earlier skill acquisition, objective performance assessment, and safer progression along the learning curve.

Several limitations should be considered when interpreting the findings of this meta-analysis. First, substantial heterogeneity was observed for Grade 1 screw placement and overall procedural success, which likely reflects variability across studies in terms of VR simulator platforms, training duration and intensity, trainee experience levels, anatomical regions, and assessment methodologies. Although random-effects models and leave-one-out sensitivity analyses were performed, the small number of included studies precluded reliable subgroup analyses or meta-regression to further investigate potential sources of heterogeneity. Second, the included trials primarily evaluated technical performance in simulated or cadaveric environments rather than in live surgical settings, limiting the direct extrapolation of these findings to real-world clinical practice and patient outcomes. The outcomes assessed in the included trials were technical or procedural measures rather than patient-centered clinical outcomes. Third, differences in pedicle screw grading systems, definitions of procedural success, and thresholds for cortical breach across studies may have influenced the pooled effect estimates and contributed to residual heterogeneity. Fourth, the relatively small number of available RCTs and limited sample sizes within individual studies may reduce the precision and generalizability of the effect estimates and limit our ability to explore potential effect modifiers through subgroup analyses. Fifth, publication bias cannot be excluded, particularly given the small number of included studies, which also limits the reliability of formal statistical approaches for its detection. Therefore, improvements in simulated technical performance should not necessarily be interpreted as evidence of improved surgical outcomes or patient care. These limitations highlight the need for future multicenter RCTs with standardized VR training protocols, uniform outcome definitions, and the inclusion of clinically relevant, patient-centered endpoints to more definitively establish the role of VR simulation in spine surgery education.

## Conclusions

This systematic review and meta-analysis demonstrated that VR–based simulation training significantly improves resident and early-career surgeons’ performance in pedicle screw placement. VR training was associated with higher rates of optimal screw placement (Grade I) and overall procedural success, alongside consistently lower rates of Grade II, III, and IV placements, indicating meaningful reductions in minor, moderate, and severe cortical breaches. Additionally, residents and early-career surgeons trained with VR exhibited shorter screw penetration distances and fewer displaced screws per resident compared with those trained using conventional methods without VR. Collectively, these findings suggest that VR simulation not only enhances technical accuracy but also mitigates the risk of potentially harmful screw misplacement, supporting its role as an effective adjunct in resident training for pedicle screw insertion.

## Supplementary Information

Below is the link to the electronic supplementary material.

Supplementary Material 1

Supplementary Material 2

## Data Availability

All data supporting the findings of this study are available within the paper and its Supplementary Information.
