# Supplementary material for: Virtual reality surgical simulator training of residents and early-career surgeons for pedicle screw placement: a systematic review and meta-analysis
Source: Eur J Orthop Surg Traumatol. 2026 Oct 27;36(1):430. doi: 10.1007/s00590-026-05008-1 (PMC13714067; doi:10.1007/s00590-026-05008-1)
Supplement: Supplementary file 1 [file 590_2026_5008_MOESM1_ESM.pdf]

VR simulator training compared to Traditional training for pedicle screw placement

Bibliography:

Certainty assessment							Summary of findings				
							Study event rates (%)			Anticipated absolute effects	
Participants (studies) Follow-up	Risk of bias	Inconsistency	Indirectness	Imprecision	Publication bias	Overall certainty of evidence	With Traditional training	With VR simulator training	Relative effect (95% CI)	Risk with Traditional training	Risk difference with VR simulator training
**Grade I screw placement**											
914 (6 RCTs)	serious[a]	serious[b]	serious[c]	not serious	none	⨁◯◯◯ Very low[a,b,c]	269/457 (58.9%)	365/457 (79.9%)	**RR 1.36** (1.15 to 1.61)	269/457 (58.9%)	**212 more per 1.000** (from 88 more to 359 more)
**Grade II screw placement**											
914 (6 RCTs)	serious[a]	not serious	serious[c]	not serious	none	⨁⨁◯◯ Low[a,c]	85/457 (18.6%)	54/457 (11.8%)	**RR 0.61** (0.45 to 0.85)	85/457 (18.6%)	**73 fewer per 1.000** (from 102 fewer to 28 fewer)
**Grade III screw placement**											
434 (5 RCTs)	serious[a]	not serious	serious[c]	not serious	very strong association	⨁⨁⨁⨁ High[a,c]	33/217 (15.2%)	0/217 (0.0%)	**RR 0.07** (0.02 to 0.30)	33/217 (15.2%)	**141 fewer per 1.000** (from 149 fewer to 106 fewer)
**Grade IV screw placement**											
354 (4 RCTs)	serious[a]	not serious	serious[c]	not serious	very strong association	⨁⨁⨁⨁ High[a,c]	8/177 (4.5%)	0/177 (0.0%)	**RR 0.16** (0.03 to 0.90)	8/177 (4.5%)	**38 fewer per 1.000** (from 44 fewer to 5 fewer)
**Pedicle screw placement success**											
914 (6 RCTs)	serious[a]	serious[b]	serious[c]	not serious	none	⨁◯◯◯ Very low[a,b,c]	354/457 (77.5%)	416/457 (91.0%)	**RR 1.17** (1.04 to 1.32)	354/457 (77.5%)	**132 more per 1.000** (from 31 more to 248 more)
**Screw penetration distance**											
300 (3 RCTs)	serious[a]	not serious	serious[c]	not serious	none	⨁⨁◯◯ Low[a,c]	150	150	-	150	MD **1.07 mm lower** (1.18 lower to 0.96 lower)
**Displaced screws per resident**											
36 (3 RCTs)	serious[a]	not serious	serious[c]	not serious	none	⨁⨁◯◯ Low[a,c]	18	18	-	18	MD **2.31 screws lower** (3.15 lower to 1.47 lower)

CI: confidence interval; **MD:** mean difference; **RR:** risk ratio

Explanations

a. All studies have some concerns about the risk of bias.
b. High heterogeneity.
c. The studies assessed pedicle screw placement in simulated or cadaveric settings rather than in clinical patients, limiting the direct applicability of the evidence to real-world surgical practice.
