# Supplementary material for: Virtual reality surgical simulator training of residents and early-career surgeons for pedicle screw placement: a systematic review and meta-analysis
Source: Eur J Orthop Surg Traumatol. 2026 Oct 27;36(1):430. doi: 10.1007/s00590-026-05008-1 (PMC13714067; doi:10.1007/s00590-026-05008-1)
Supplement: Supplementary file 2 [file 590_2026_5008_MOESM2_ESM.docx]

**Supplementary Material**

**Table S1.** Definitions of pedicle screw placement accuracy

| **Class** | **Definitions** |
| --- | --- |
| Class I | Class I represented ideal placement, defined by complete containment of the screw within the cortical boundaries of the pedicle. This included descriptions such as “no penetration of the pedicle wall,” “no perforation,” “entire screw placed within the cortical bone without breaching the anterior vertebral wall,” “screw completely inside the pedicle,” and corresponded to Gertzbein–Robbins Grade A. |
| Class II | Class II encompassed minor cortical breaches, characterized by pedicle wall penetration of ≤2 mm, or violations involving less than 25–50% of the screw diameter without anterior vertebral wall involvement. These definitions aligned with terms such as “pedicle wall penetration ≤2 mm,” “minor perforation,” and “Grade 1B” in some classifications, corresponding to minor deviations within the Gertzbein–Robbins Grade B range. |
| Class III | Class III represented moderate breaches, defined as pedicle wall violations of 2–4 mm, or violations involving 25–50% of the screw diameter, sometimes described as “major perforation,” or “breakthrough without protruding the anterior vertebral wall.” These criteria aligned with Gertzbein–Robbins Grade C, depending on the extent of violation. |
| Class IV | Class IV included severe breaches, defined as pedicle wall penetration of ≥4 mm, or violations involving more than 50% of the screw diameter, including cortical breakthrough with anterior vertebral wall protrusion. This category corresponded to the most severe classifications within the Gertzbein–Robbins Grades D-E spectrum. |

**Figure S1.** Leave-one-out sensitivity analysis plot of the pedicle screw placement success outcome.

**Figure S2.** Critical appraisal of randomized controlled trials according to the Cochrane Collaboration’s tool for assessing risk of bias in randomized trials.
